# Genome-wide association study on resistance of cultivated soybean to *Fusarium oxysporum* root rot in Northeast China

**DOI:** 10.1186/s12870-023-04646-5

**Published:** 2023-12-07

**Authors:** Yongsheng Sang, Xiaodong Liu, Cuiping Yuan, Tong Yao, Yuqiu Li, Dechun Wang, Hongkun Zhao, Yumin Wang

**Affiliations:** 1 Soybean Research Institute, Jilin Academy of Agricultural Sciences, National Engineering Research Center for Soybean, Changchun, 130118 Jilin PR China; 2 College of Agronomy, Jilin Agricultural University, Changchun, 130118 Jilin PR China; 3 Crop Germplasm Institute, Jilin Academy of Agricultural Sciences, Changchun, 130118 Jilin China; 4 Department of Plant, Soil and Microbial Sciences, Michigan State University, 1066 Bogue St., Rm. A384-E, East Lansing, MI 48824 USA

**Keywords:** Soybean, *Fusarium oxysporum* root rot, Single nucleotide polymorphism, GWAS, Candidate genes

## Abstract

**Background:**

*Fusarium oxysporum* is a prevalent fungal pathogen that diminishes soybean yield through seedling disease and root rot. Preventing *Fusarium oxysporum* root rot (FORR) damage entails on the identification of resistance genes and developing resistant cultivars. Therefore, conducting fine mapping and marker development for FORR resistance genes is of great significance for fostering the cultivation of resistant varieties. In this study, 350 soybean germplasm accessions, mainly from Northeast China, underwent genotyping using the SoySNP50K Illumina BeadChip, which includes 52,041 single nucleotide polymorphisms (SNPs). Their resistance to FORR was assessed in a greenhouse. Genome-wide association studies utilizing the general linear model, mixed linear model, compressed mixed linear model, and settlement of MLM under progressively exclusive relationship models were conducted to identify marker-trait associations while effectively controlling for population structure.

**Results:**

The results demonstrated that these models effectively managed population structure. Eight SNP loci significantly associated with FORR resistance in soybean were detected, primarily located on Chromosome 6. Notably, there was a strong linkage disequilibrium between the large-effect SNPs ss715595462 and ss715595463, contributing substantially to phenotypic variation. Within the genetic interval encompassing these loci, 28 genes were present, with one gene *Glyma.06G088400* encoding a protein kinase family protein containing a leucine-rich repeat domain identified as a potential candidate gene in the reference genome of Williams82. Additionally, quantitative real-time reverse transcription polymerase chain reaction analysis evaluated the gene expression levels between highly resistant and susceptible accessions, focusing on primary root tissues collected at different time points after *F. oxysporum* inoculation. Among the examined genes, only this gene emerged as the strongest candidate associated with FORR resistance.

**Conclusions:**

The identification of this candidate gene *Glyma.06G088400* improves our understanding of soybean resistance to FORR and the markers strongly linked to resistance can be beneficial for molecular marker-assisted selection in breeding resistant soybean accessions against *F. oxysporum*.

**Supplementary Information:**

The online version contains supplementary material available at 10.1186/s12870-023-04646-5.

## Background

Cultivated soybean (*Glycine max* (L.) Merrill) holds great significance globally as a major oil crop, owing to its remarkable ability to enhance human nutrition as a valuable source of food, protein, and oil [1]. However, soybeans are highly susceptible to numerous pathogens, leading to substantial losses in production and quality deterioration [2, 3]. Among these pathogens, *Fusarium oxysporum Schltdl* is a particularly troublesome soil-borne fungal disease, causing soybean root rot and inflicting significant economic losses on soybean production. In the initial stages of the disease, the color of the root tip undergoes a visible change, eventually giving rise to dark plaques on the main root. These diseased lesions persist without shrinking, gradually expanding and accompanied by cortex blackening, ultimately leading to rot and necrosis [4].

The “*F. oxysporum* species complex” is a globally distributed soil-borne fungal pathogen that exhibits facultative parasitic behavior [5, 6]. It is recognized as one of the most prevalent and aggressive *Fusarium* spp. responsible for soybean root rot in Southwest, Northeast China, and North America [4, 7]. *F. oxysporum* colonizes the vascular system of plant roots and employs various secretion mechanisms, as well as produces a variety of virulence factors such as mycotoxins, effector proteins, and plant cell wall degrading enzymes. These factors play a crucial role in the destruction of xylem vessels responsible for water transport, resulting in wilt symptoms and eventual plant death [8, 9]. Managing soil-borne diseases like *Fusarium* root rot primarily relies on genetic resistance and seed treatment [10]. Although seed treatment is effective during the seedling stage, relying solely on a single agent may lead to drug-resistant pathogens and inadequate control in later stages of plant growth. Breeding resistant varieties emerges as the most practical, cost-effective, and environmentally friendly approach to long-term pathogen management [11]. The complex nature of disease resistance in plants is influenced by both genetic and environmental factors. Identifying the genes responsible for conferring resistance is crucial in developing disease-resistant crops. Molecular breeding necessitates a comprehensive understanding of the genetic basis underlying complex traits. In contrast to the utilization of biparental linkage mapping for identifying loci associated with disease resistance, genome-wide association studies (GWAS) have emerged as a powerful method to establish connections between molecular markers, such as single nucleotide polymorphisms (SNPs), and phenotypic traits, owing to the utilization of high-density and high-quality marker data [12]. By leveraging historical recombination events on a population level, GWAS offer a valuable approach to overcome the limitations imposed by studying traits in only two individuals. This enables high-resolution mapping of the genes linked to complex traits [13]. In comparison to linkage analysis using biparental mapping populations, GWAS has the potential to significantly enhance the resolution and accuracy of marker-phenotype relationships. In soybean, GWAS has successfully identified markers associated with diverse disease resistance traits, including sudden death syndrome [12, 14, 15], soybean cyst nematode resistance [12, 16, 17], soybean mosaic virus [12, 18], Sclerotinia stem rot [19–21], white mode [22–24], Phytophthora root rot [12, 25], brown stem rot [12, 26], root knot nematode [27] and southern root knot nematode [28], bacterial pustule, bean pod mottle virus, Diaporthe stem canker, peanut mottle virus, reniform nematode, and soybean rust. However, GWAS mapping has not been employed to investigate traits related to *Fusarium oxysporum* root rot (FORR) resistance in soybeans. Therefore, the objectives of this study were twofold: (i) to employ GWAS to identify loci associated with FORR resistance and (ii) to investigate differential expression to determine candidate genes for the identified loci. These findings can enhance our understanding of the genetic basis of FORR resistance in cultivated soybeans, facilitating the diversification of resistance genes in cultivars, and enabling the identification and application of markers adjacent to resistance genes in marker-assisted selection (MAS).

## Results

### Greenhouse evaluation for FORR resistance

**Fig. 1:**
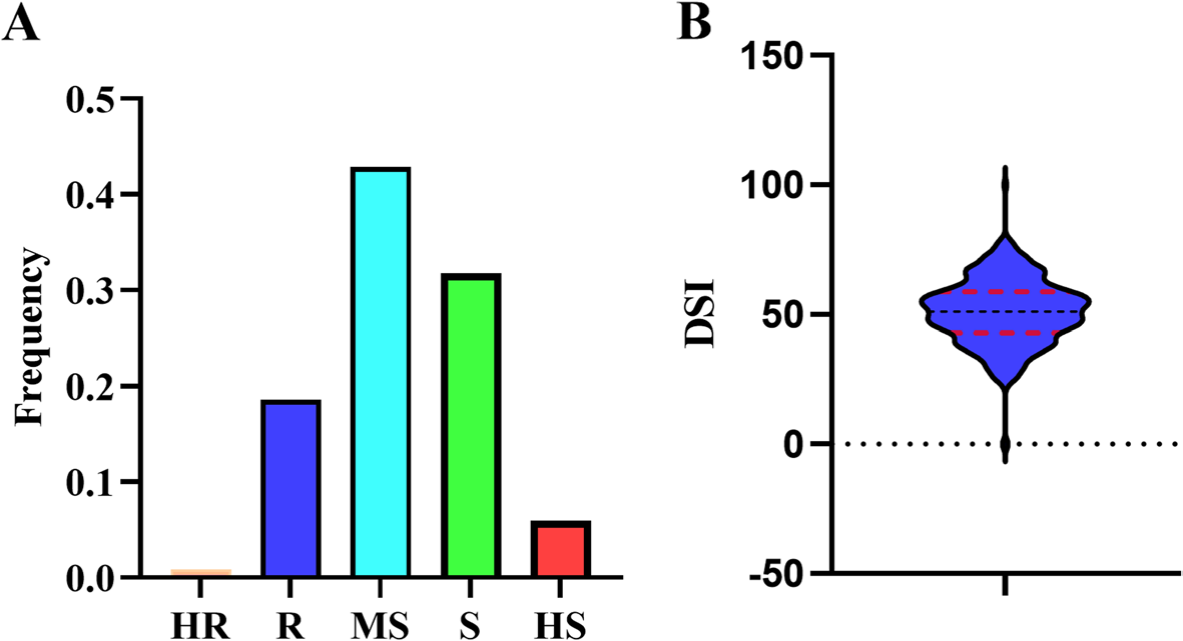
Disease severity index (DSI) of 350 SGAs exposed to *F. oxysporum*. **A** Bio-assay results of the FORR resistance in 350 SGAs. **B** Phenotypic distribution of FORR resistance in 350 SGAs

Within the association mapping population, a wide range of phenotypic variation in FORR resistance was observed, as measured by the disease severity index (DSI) (DSI, see Additional file 1: Table S1). Among the 350 evaluated soybean germplasm accessions (SGAs), the DSI values spanned from 0 to 100, with an average of 51.01. The DSI values exhibited a continuous distribution, adhering to a reverse normal distribution pattern (W = 0.98669, *p*-value = 0.002641), and there was no serious non-normal distribution (Additional File 2: Figure S1). Out of the 350 SGAs, 21 were classified as highly susceptible (HS), 111 as susceptible (S), 150 as moderately susceptible (MS), 65 as resistant (R), and 3 as highly resistant (HR) (Fig. 1A and B).

### Quality control, distribution of SNPs, and linkage disequilibrium decay

**Fig. 2:**
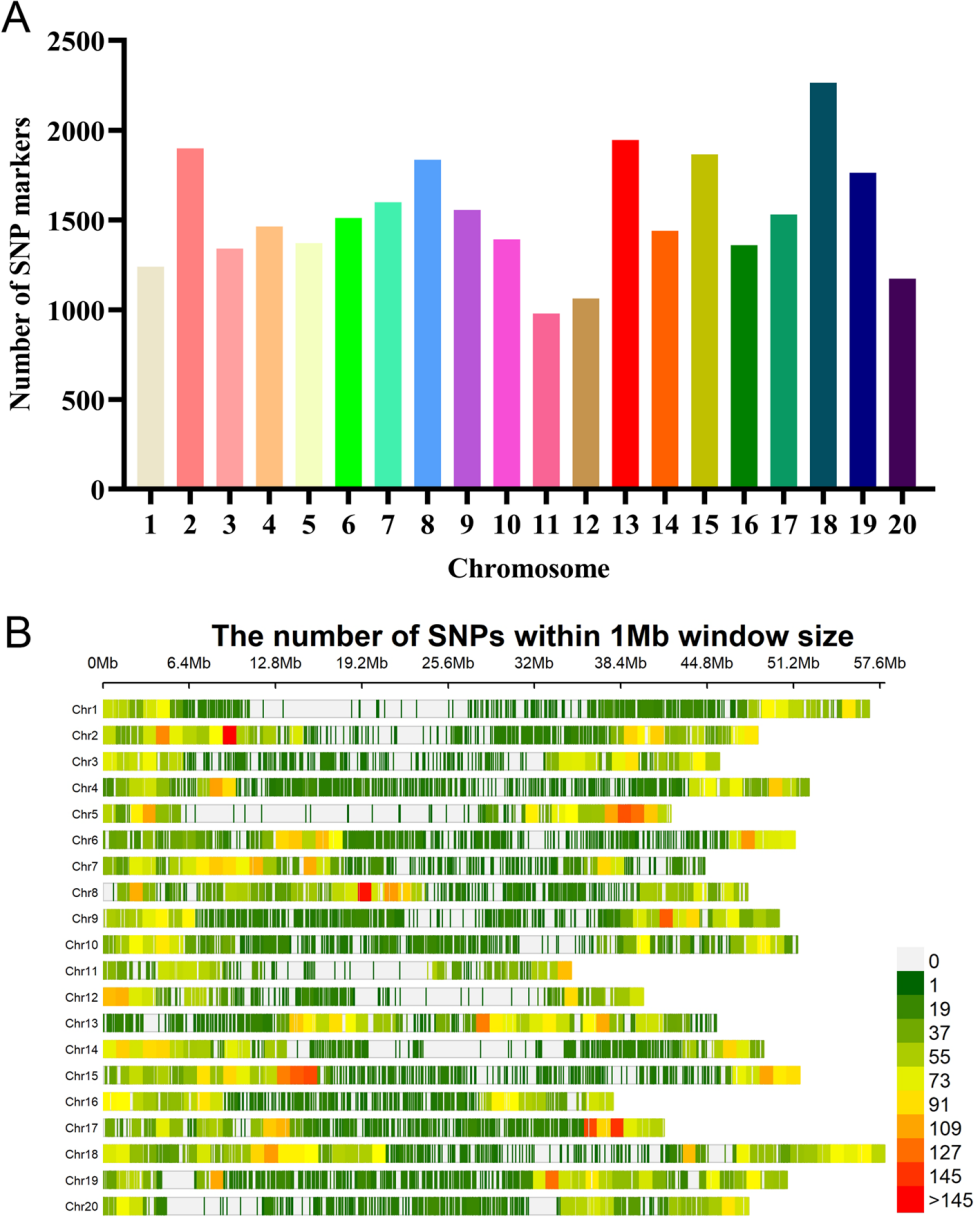
Distribution analysis of 30,602 SNPs across the 350 SGAs. **A** Distribution of SNP markers across 20 soybean chromosomes. **B** Density of SNP markers per chromosome. The horizontal axis displays the chromosome length (Mb), and the vertical axis indicates the chromosome number, with SNP density displayed in various colors

**Fig. 3:**
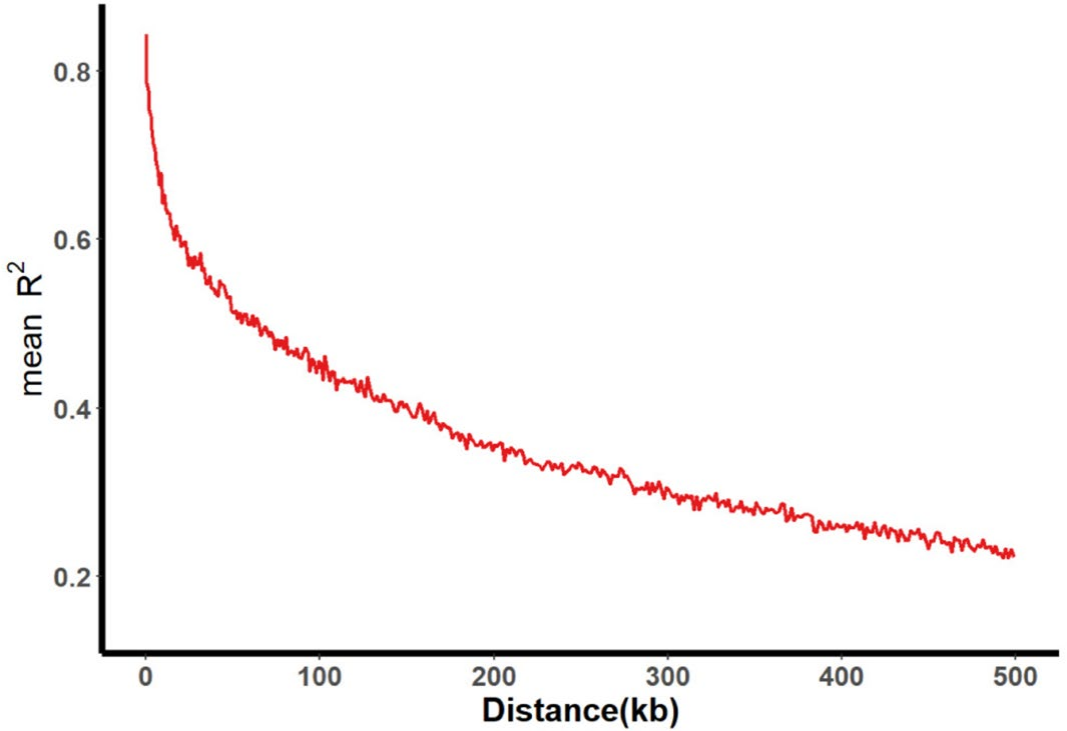
Average linkage disequilibrium (LD) decay rate of the soybean genome. The mean LD decay rate was estimated as *r*^2^ using all SNP pairs within a 500 kb physical distance in euchromatic and heterochromatic regions of 350 SGAs

Genotyping of 350 SGAs was conducted using the SoySNP50K BeadChip, resulting in the characterization of profiles for 52,041 SNPs. After the initial filtering process, 30,602 SNPs were retained by excluding those with minor allele frequencies of less than 5% in at least 80% of the soybean genotypes. These high-quality SNP markers provided comprehensive coverage across the genome, with a genome-wide average SNP marker density of 31.25 kb/SNP. Among the chromosomes (Chr), Chr 20 exhibited the highest SNP density (15.06 kb/SNP), while Chr 13 had the lowest SNP density (42.42 kb/SNP). The distribution of SNPs throughout the soybean genome was irregular, with an average of 1530 SNPs per Chr. Chr 18 had the highest number of SNPs (2265), whereas Chr 11 had the lowest (980) (Additional File 3: Table S2, Fig. 2A and B). In addition, the association panel was used to assess genome-wide linkage disequilibrium (LD) decay. A noticeable decline in LD was observed as the physical distance between paired SNPs increased. Pairwise LD was estimated within a 500 kb window, and the LD decay rate, measured by the point at which the correlation coefficient (*r*^2^) dropped to half of its maximum value, was determined to be 109 kb at *r*^2^ = 0.422 (Fig. 3). The observed LD decay was lower compared to previously reported values for landraces (187 kb) and improved lines (233 kb) [29]. This discrepancy may be attributed to the smaller number of genotypes included in the two panels, as the same BeadChip was used for genotyping.

### Analysis of population structure of 350 SGAs

**Fig. 4:**
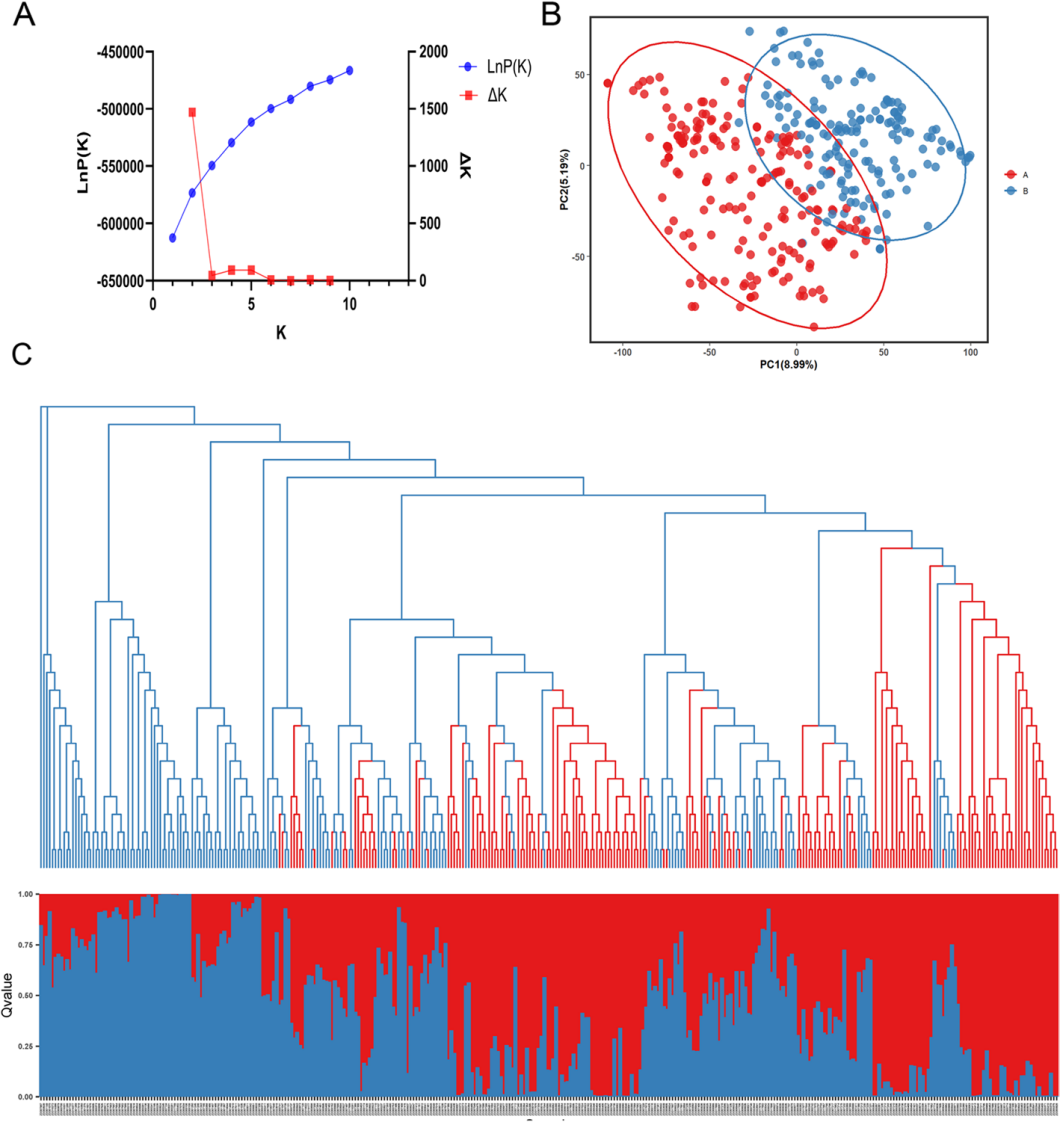
Population structure analysis of 350 soybean germplasm accessions. **A** Mean LnP(k) and Delta k values for k ranging from 1 to 10. **B** Two-dimensional PCA scatter plot, with red dots representing subgroup 1 and blue dots representing subgroup 2. **C** Combined map of population structure and neighbor-joining tree for 350 SGAs, displaying the percentage of individuals in each segment. Note: Grouped by population structure

The population structure of the 350 SGAs was assessed based on 1643 unlinked SNPs using STRUCTURE 2.3.4 software [30]. Analysis of the Delta K value revealed a prominent peak at K = 2 (Fig. 4A), indicating the presence of two sub-populations, referred to as cluster Q1 and Q2. The SGAs originated from various geographical regions including Heilongjiang province (HLJ), Jilin province (JL), Liaoning province (LN), Inner Mongolia (IM), Beijing (BJ), and Shanxi province (SX). Among the 350 SGAs, 190 were assigned to the Q1 sub-population, including 61 from HLJ, 100 from JL, 28 from LN, and 1 from SX. The Q2 sub-population comprised 160 SGAs, with 62 from HLJ, 57 from JL, 33 from LN, 7 from IM, and 1 from BJ (Additional File 4: Table S3, Fig. 4C). A Q-matrix, obtained when the optimal K value was determined, was employed for association mapping. Principal component analysis (PCA) and the phylogenetic tree analysis of the 350 SGAs confirmed the clustering patterns predicted by the STRUCTURE analysis (Fig. 4B and C). These findings indicated the presence of a subpopulation structure within the 350 SGAs, and the Q matrix could serve as a covariate in the GWAS model to reduce the false-positive rate.

### Model comparison for the control of false associations

**Fig. 5:**
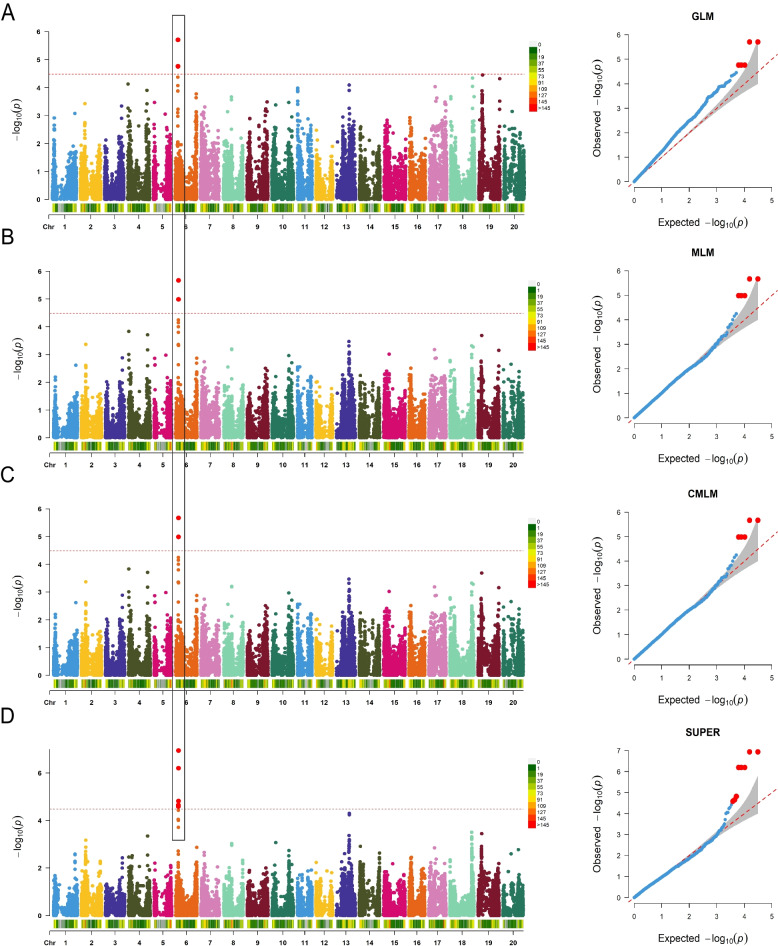
Genome-wide association study of FORR in 350 SGAs. **A** Manhattan plot and quantile–quantile (Q-Q) plot from the genome-wide analysis of FORR resistance using GLM(Q). Chr numbers are represented on the horizontal axis, and the -log10 *P*-values are represented on the vertical axis. The horizontal red dotted line indicates the Bonferroni test threshold as 1/total (-log_10_*P* = 4.49). **B** Manhattan and Q-Q plots from the genome-wide analysis of FORR resistance using MLM (Q + K). **C** Manhattan and Q-Q plots from the genome-wide analysis of FORR resistance using CMLM (Q + K). (D) Manhattan and Q-Q plots from the genome-wide analysis of FORR resistance using SUPER (Q + K)

To minimize false positive marker-trait associations caused by population structure and relatedness, the statistical models considered both the population structure matrix (Q) and kinship matrix (K) in the GWAS analysis [31]. For the investigation of FORR resistance, association mapping was performed while controlling for false associations using Q, K, and PCA. To identify a significant SNP associated with DSI, SNP-trait associations were investigated using four models: general linear model (GLM), mixed linear model (MLM), compressed mixed linear model (CMLM), and settlement of MLM under progressively exclusive relationship (SUPER). Comparison of the quantile–quantile plots (Q-Q plots) of the GLM (Q), MLM (Q + K), CMLM (Q + K), and SUPER (Q + K) models revealed that the observed *P*-values in the MLM (Q + K), CMLM (Q + K), and SUPER (Q + K) models closely matched the expected *P*-values initially. However, as the -log10 *P* value increased to approximately 3.3, the observed *P*-values deviated significantly from the expected P-values (Fig. 5B–D). This indicated that the three models effectively controlled false-positive associations and avoided false-negative correlations. In contrast, the observed *P*-values of the GLM (Q) model were consistently higher than the expected values, indicating poor control of false positives and overestimation of significance (Fig. 5A). Hence, the MLM (Q + K), CMLM (Q + K), and SUPER (Q + K) models were selected for association mapping in this study.

### Genome wide association analysis for FORR Resistance

The significance threshold for the Bonferroni correction line employed in this study was determined as -log10(1/30,602) = 4.49. Eight SNPs (ss715595416-281.0kb-ss715595451-61.4kb-ss715595455-0.2kb-ss715595456-67.8kb-ss715595462-9.1kb-ss715595463-24.4kb-ss715595465-24.5kb-ss715595467) significantly associated with DSI were identified from the greenhouse evaluations (Table 1). All of these SNPs were situated on Chr 06, encompassing a 468.4 kb region ranging from 6441.5 kb to 6909.9 kb. They accounted for 4.58%–6.95% of the phenotypic variation in FORR resistance. Among the four GWAS models, ss715595462 and ss715595463 exhibited the highest significance, followed by ss715595455, ss715595456, and ss715595465. These two large-effect SNPs (ss715595462 and ss715595463) on Chr 06 were considered to be crucial loci that can be used to explore potential candidate genes associated with FORR resistance in this study.

**Table 1:** Significant SNPs detected by different statistic models

Method	SNP	Physical position		Significant region		-log10 (P)	MAF	PVE (phenotypic variance) (%)
		Chr	Position	Start	End			
GLM	ss715595462	6	6,851,950	6,742,950	6,960,950	5.70	0.09	6.70%
GLM	ss715595463	6	6,861,034	6,752,034	6,970,034	5.70	0.09	6.70%
GLM	ss715595456	6	6,784,111	6,675,111	6,893,111	4.76	0.11	5.44%
GLM	ss715595465	6	6,885,409	6,776,409	6,994,409	4.76	0.11	5.44%
GLM	ss715595455	6	6,783,932	6,674,932	6,892,932	4.76	0.11	5.44%
MLM	ss715595463	6	6,861,034	6,752,034	6,970,034	5.67	0.09	6.63%
MLM	ss715595462	6	6,851,950	6,742,950	6,960,950	5.67	0.09	6.63%
MLM	ss715595455	6	6,783,932	6,674,932	6,892,932	4.99	0.11	5.71%
MLM	ss715595456	6	6,784,111	6,675,111	6,893,111	4.99	0.11	5.71%
MLM	ss715595465	6	6,885,409	6,776,409	6,994,409	4.99	0.11	5.71%
CMLM	ss715595463	6	6,861,034	6,752,034	6,970,034	5.67	0.09	6.63%
CMLM	ss715595462	6	6,851,950	6,742,950	6,960,950	5.67	0.09	6.63%
CMLM	ss715595455	6	6,783,932	6,674,932	6,892,932	4.99	0.11	5.71%
CMLM	ss715595456	6	6,784,111	6,675,111	6,893,111	4.99	0.11	5.71%
CMLM	ss715595465	6	6,885,409	6,776,409	6,994,409	4.99	0.11	5.71%
SUPER	ss715595463	6	6,861,034	6,752,034	6,970,034	6.95	0.09	NA
SUPER	ss715595462	6	6,851,950	6,742,950	6,960,950	6.95	0.09	NA
SUPER	ss715595456	6	6,784,111	6,675,111	6,893,111	6.20	0.11	NA
SUPER	ss715595465	6	6,885,409	6,776,409	6,994,409	6.20	0.11	NA
SUPER	ss715595455	6	6,783,932	6,674,932	6,892,932	6.20	0.11	NA
SUPER	ss715595467	6	6,909,916	6,800,916	7,018,916	4.82	0.11	NA
SUPER	ss715595416	6	6,441,486	6,332,486	6,550,486	4.64	0.07	NA
SUPER	ss715595451	6	6,722,530	6,613,530	6,831,530	4.58	0.06	NA

### Prediction of candidate genes

**Fig. 6:**
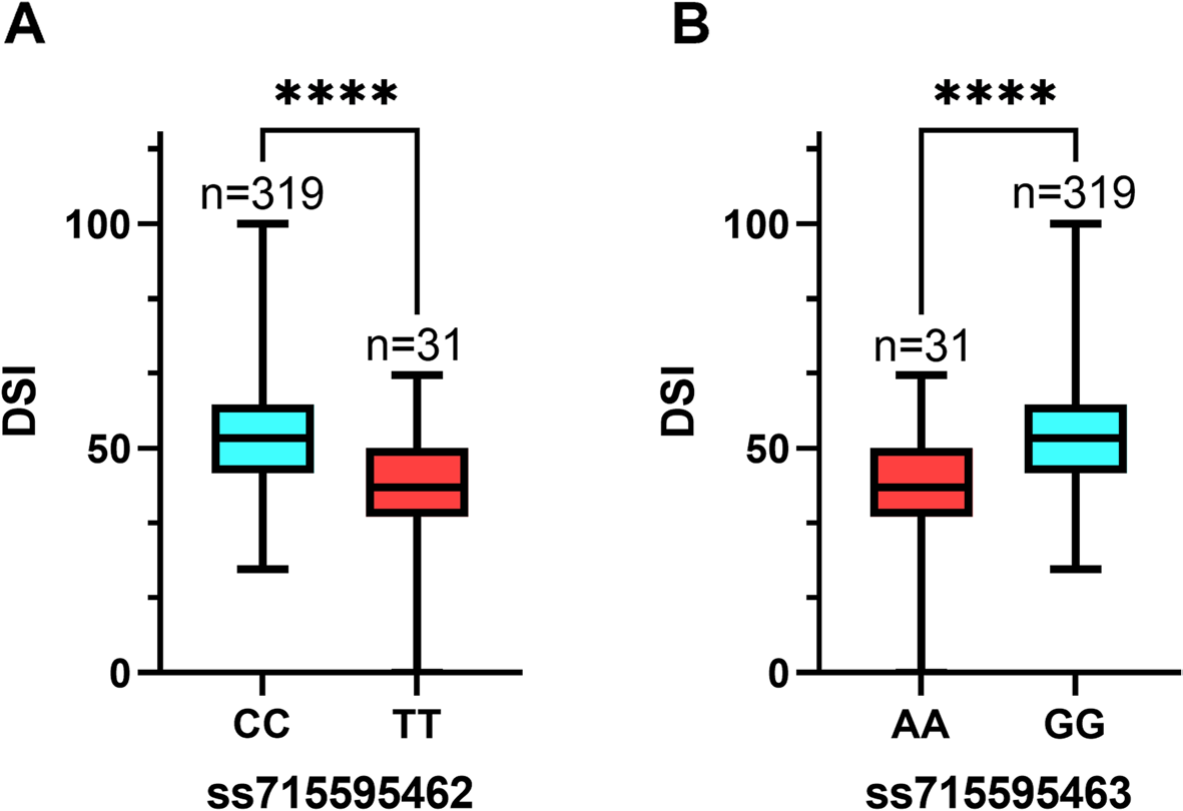
Differences in FORR resistance among accessions with different alleles. **A** Allele effects for the FORR marker ss715595462 in the 350 SGAs. **B** Allele effects for the FORR marker ss715595463 in the 350 SGAs

**Fig. 7:**
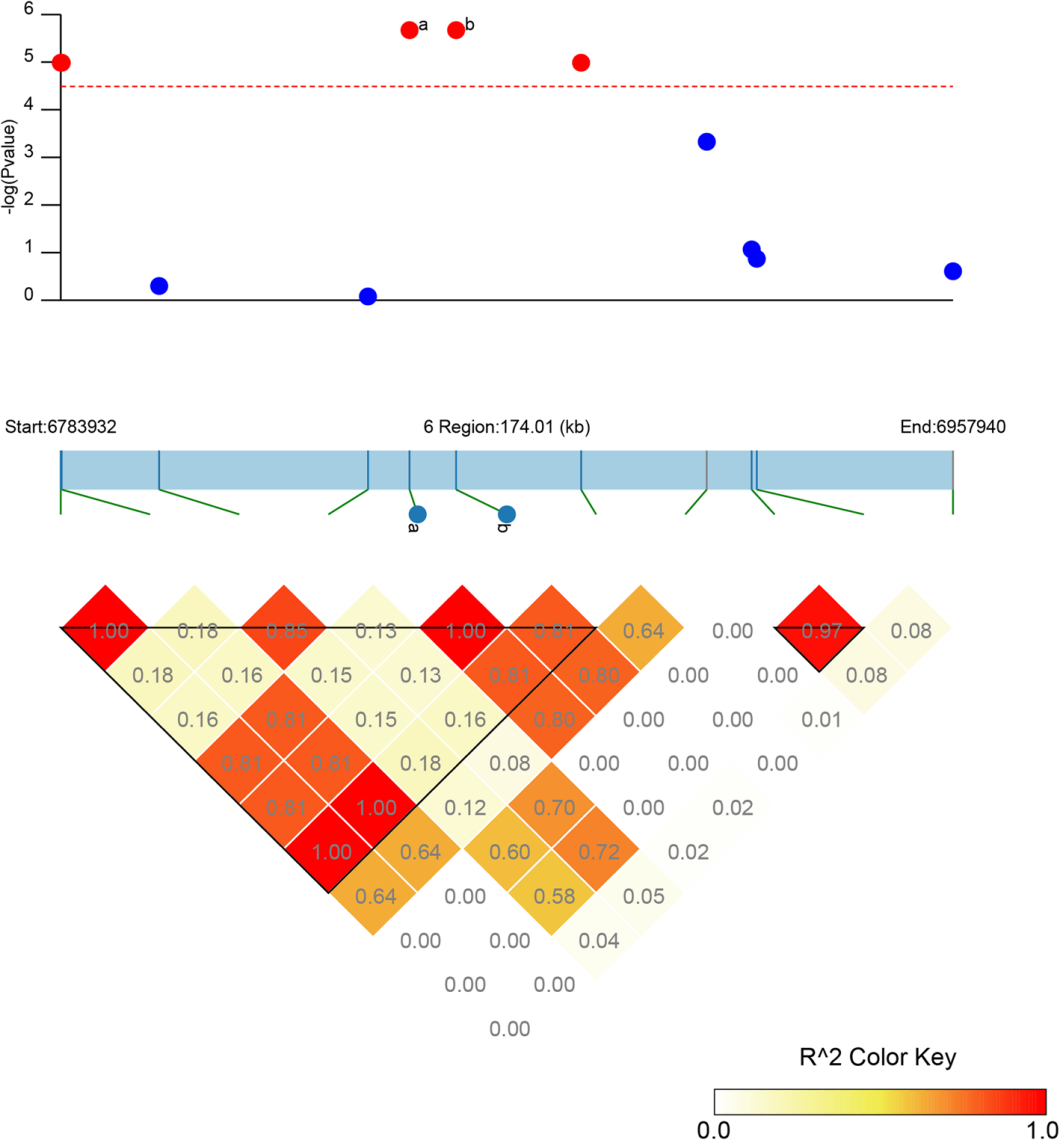
Candidate regions of large-effect QTNs associated with soybean FORR resistance. The top panel displays the -log10 *P*-values of the SNP from the FORR of GWAS, plotted against their physical location of a given chromosome region. The bottom panel depicts the horizontal LD range based on *R*^2^ values, and the color key displays *R*^2^ values. The horizontal red dotted line represents the significance threshold for GWAS (-log10(*p*) > 4.49). Note: **a** significant SNP ss715595462; **b** significant SNP ss715595463

We identified eight significant SNPs on Chr 06, with particular interest in the large-effect SNPs ss715595462 (Gm06_6851950, MAF = 0.09) and ss715595463 (Gm06_6861034, MAF = 0.09). These SNPs contributed the most to the phenotypic variation, as evidenced by their average DSI of 40.52, which was significantly lower than the mean DSI of the entire panel (51.01). The soybean plants carrying the favorable allele (TT) on ss715595462 exhibited noticeably higher FORR resistance (mean DSI = 40.52) compared to those carrying the unfavorable allele (CC) (mean DSI = 52.03) (Fig. 6A). Similarly, soybeans carrying the favorable allele (AA) on ss715595463 showed significantly higher FORR resistance (mean DSI = 40.52) compared to those carrying the adverse allele (GG) (mean DSI = 52.03) (Fig. 6B). LD analysis revealed a strong LD between the large-effect SNPs ss715595462 and ss715595463 loci (Fig. 7). Therefore, we focused on a 227.1 kb region spanning from 109 kb before ss715595462 to 109 kb after ss715595463 and predicted candidate genes using the gene model of the soybean genome assembly version Glyma.Wm82.a2.v1. Within this region, we discovered 28 putative genes (*Glyma.06g087100*–*Glyma.06g089800*), with only one gene containing leucine-rich repeat receptor-like kinase (LRR-RLK), which plays a crucial role in plants’ response to various external stimuli, including pathogens (Table 2).

**Table 2:** Annotation of candidate genes in the loci linkage region of Chr 06 for the two significant SNPs (ss715595462 and ss715595463)

NO	Gene ID^a^	Ortholog^b^	Fuction annotation
1	*Glyma.06G087100*	AT5G11650.1	alpha/beta-Hydrolases superfamily protein
2	*Glyma.06G087200*	AT4G32870.1	Polyketide cyclase/dehydrase and lipid transport superfamily protein
3	*Glyma.06G087300*	AT4G32870.1	Polyketide cyclase/dehydrase and lipid transport superfamily protein
4	*Glyma.06G087400*	AT4G32860.1	unknown protein
5	*Glyma.06G087500*	AT2G25760.1	Protein kinase family protein
6	*Glyma.06G087600*	NA	NA
7	*Glyma.06G087700*	AT1G15215.2	sequence-specific DNA binding transcription factors;sequence-specific DNA binding
8	*Glyma.06g087800*	AT5G25880.1	NADP-malic enzyme 3
9	*Glyma.06G087900*	AT2G25740.1	ATP-dependent protease La (LON) domain protein
10	*Glyma.06G088000*	AT2G25840.2	Nucleotidylyl transferase superfamily protein
11	*Glyma.06G088100*	AT3G16360.2	HPT phosphotransmitter 4
12	*Glyma.06G088200*	AT1G10010.1	amino acid permease 8
13	*Glyma.06G088300*	AT1G10010.1	amino acid permease 8
14	*Glyma.06G088400*	AT5G25930.1	Protein kinase family protein with leucine-rich repeat domain (LRR-RLK)
15	*Glyma.06G088500*	AT4G32850.9	nuclear poly(a) polymerase
16	*Glyma.06G088600*	AT4G26270.1	phosphofructokinase 3
17	*Glyma.06G088700*	AT2G25880.2	ataurora2/a member of a family of Ser/Thr kinases
18	*Glyma.06G088800*	AT4G32830.1	ataurora1/a family of Ser/Thr kinases
19	*Glyma.06G088900*	AT4G32820.1	Tetratricopeptide repeat (TPR)-like superfamily protein
20	*Glyma.06G089000*	AT5G11700.2	unknown protein
21	*Glyma.06G089100*	AT1G10040.1	alpha/beta-Hydrolases superfamily protein
22	*Glyma.06G089200*	AT4G37810.1	unknown protein
23	*Glyma.06G089300*	AT2G22795.1	unknown protein
24	*Glyma.06G089400*	AT1G10030.1	homolog of yeast ergostero
25	*Glyma.06G089500*	NA	NA
26	*Glyma.06G089600*	AT1G10020.1	Protein of unknown function (DUF1005)
27	*Glyma.06G089700*	AT5G17870.1	plastid-specific 50S ribosomal protein 6
28	*Glyma.06G089800*	AT4G08170.2	Inositol 1,3,4-trisphosphate 5/6-kinase family protein

^a^Glyma ID from soybean reference genome Wm82.a2.v1 (http://www.soybase.org)

^b^Accession numbers for Arabidopsis orthologs are from the Arabidopsis Information Resource (TAIR10, http://www.arabidopsis.org/)

To gain further insights into the potential functions of these genes, we performed a comprehensive analysis using GO enrichment analysis (http://bioinfo.cau.edu.cn/agriGO) and KOG functional annotation (http://phytozome.jgi.doe.gov/pz/portal.html#!info?alias=Org_Gmax). This classification and annotation approach allowed us to assign the genes into different functional groups based on their putative roles. We observed that *Glyma.06g088400* exhibited associations with protein phosphorylation (GO:0006468) and phosphorylation (GO:0016310) in the biological process category of gene ontology. In the molecular function category, it was linked to protein binding (GO:0005515), transferase activity (GO:0016740), kinase activity (GO:0016301), protein serine/threonine kinase activity (GO:0004674), ATP binding (GO:0005524), protein kinase activity (GO:0004672), and nucleotide binding (GO:0000166). Furthermore, in the cellular component category, it showed associations with integral component of membrane (GO:0016021) and membrane (GO:0016020) (Additional File 5: Table S4). Additionally, we identified three protein kinase families (*Glyma.06g087500*, *Glyma.06g088700*, and *Glyma.06g088800*) with similar functional descriptions to *Glyma.06g088400*, encompassing ATP binding (GO:0005524), protein serine/threonine kinase activity (GO:0004674), protein kinase activity (GO:0004672), and protein phosphorylation (GO:0006468) in both the biological process and molecular function categories of gene ontology. These four genes (*Glyma.06g087500*, *Glyma.06g088400*, *Glyma.06g088700*, and *Glyma.06g088800*) are primarily involved in cell cycle control, signal transduction mechanisms, chromosome partitioning, and cell division through the production of serine/threonine protein kinase (Additional File 6: Table S5). Furthermore, based on the gene model of soybean genome assembly version Glyma.Wm82.a2.v1, we found that *Glyma.06g087700* functions as a sequence-specific DNA binding transcription factor, associated with DNA methylation-dependent heterochromatin formation (GO:0006346), DNA methylation (GO:0006306), and cotranscriptional gene silencing by small RNA (GO:0033562) in the biological process category of gene ontology. It is also involved in chromatin binding (GO:0003682) in the molecular function category and is localized in the nucleus (GO:0005634) according to the cellular component category (Additional File 5: Table S4). Hence, these five genes are considered as potential candidate genes, associated with resistance to *F. oxysporum*. Furthermore, we found that the functions of *Glyma.06G087400*, *Glyma.06G089000*, *Glyma.06G089200*, *Glyma.06G089300* and *Glyma.06G089600* genes remian unknown. Nevertheless, these five genes with unknown functions may also be linked to resistance against *F. oxysporum.* However, further functional validation is necessary to confirm their specific roles in disease defense mechanisms.

### Expression profiling for candidate genes

**Fig. 8:**
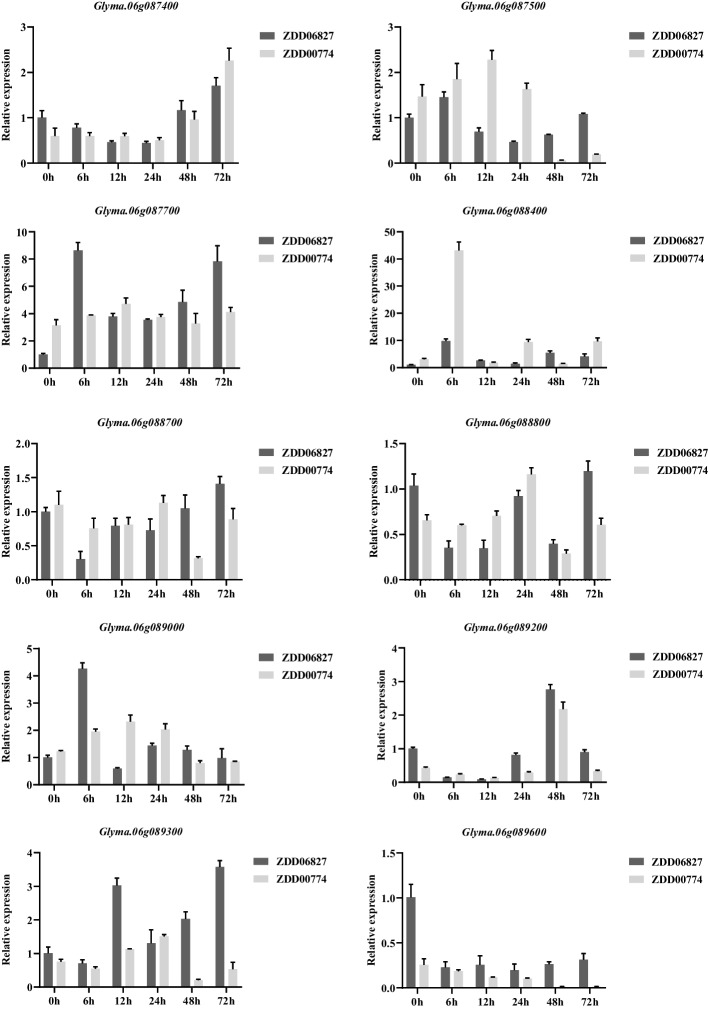
Relative expression levels of candidate genes *Rmd_ZDD00774* in ZDD06827 (HS) and ZDD00774 (HR). Values are presented as the means ± SEs (*n* ≥ 3)

To investigate the gene expression changes induced by *F. oxysporum* infection, we analyzed the expression patterns of 10 candidate genes in two soybean accessions, namely ZDD00774 (HR) and ZDD06827 (HS). These accessions carry favorable and unfavorable alleles, respectively, at the ss715595462 and ss715595463 loci. Using qRT-PCR, we found that among the 10 genes, only *Glyma.06g088400* exhibited differential expression between ZDD00774 and ZDD06827. Following treatment with *F. oxysporum*, the expression of *Glyma.06g088400* was upregulated in the highly resistant accession ZDD00774. Specifically, the expression level of *Glyma.06g088400* in ZDD00774 (HR) showed a rapid increase at 6 h after treatment, reaching a maximum fold-change of approximately 43.2. This upregulation was significantly higher compared to ZDD06827 (HS), which exhibited an approximate fold-change of 9.8 during the same period. Subsequently, the expression of *Glyma.06g088400* declined rapidly at 12, 24, 48, and 72 h after treatment (Fig. 8). These findings suggest that *Glyma.06g088400* is induced by *F. oxysporum* and may play a role in the soybean’s defense mechanism against the disease.

## Discussion

The host–pathogen interaction in the context of FORR is characterized by a highly intricate collaboration among multiple genes that trigger molecular signals and subsequent responses [16]. Understanding the loci responsible for resistance not only contributes to genetic improvement in cultivars but also facilitates the identification of resistant genes and enhances our comprehension of the molecular mechanisms underlying the resistance process [16]. Given that FORR is a complex quantitative trait, it is imperative to unravel the genetic foundations and genes implicated in this condition, as they represent a primary focus of breeding programs aimed at developing resistant soybean varieties harboring durable resistance genes/QTLs. However, the genetic mechanisms governing FORR in soybeans at the seedling stage are still inadequately understood. GWAS, which leverage ancient recombination events to uncover genetic loci associated with crop traits, have emerged as a robust technique for identifying potential natural variations related to complex traits in crops [32]. The rapid advancements in whole-genome sequencing techniques and computational methods have greatly contributed to the effectiveness of GWAS in identifying crucial genomic regions associated with traits of interest and predicting candidate genes [33, 34]. However, decoding the biological significance of GWAS signals can be challenging, as the identified loci often reside within gene deserts or regions with a multitude of potential causative genes [35]. To aid in the interpretation of GWAS results, the analysis of differential gene expression has been proposed as a promising strategy [36]. In this study, GWAS was conducted to genotype 350 SGAs using a high-quality dataset of 30,602 SNPs. The association panel used in this study exhibited a rich diversity of natural genetic variations. Through GWAS, we identified eight SNPs on Chr 06 (ranging from 6,441,486 to 6,909,916 bp) that were significantly associated with DSI for FORR resistance. To further investigate the potential candidate genes underlying FORR resistance, we conducted candidate gene prediction and performed qRT-PCR verification for the genomic regions harboring two large-effect and closely linked SNPs, namely ss715595462 and ss715595463, located on Chr 06. Interestingly, our analysis revealed that only the gene *Glyma.06g088400*, which contains LRR-RLK domain, exhibited differential expression between ZDD00774 and ZDD06827 after treatment with *F. oxysporum*. Previous studies have indicated that genes showing distinct expression patterns among different accessions are more likely to be associated, either directly or indirectly, with susceptibility or resistance outcomes. On the other hand, genes exhibiting distinct expression dynamics over time are more likely to represent the general response of plants to pathogen infection, without necessarily conferring increased resistance [37]. By referring to the *Glycine max* genome assembly (Glyma.Wm82.a2.v1), we determined that the large-effect SNP ss715595462 (Chr06_6851950, MAF = 0.09) resides within the coding region of the *Glyma.06g088400* (Chr 06_6851663–6857631). Additionally, the large-effect SNP ss715595463 (Chr 06_6861034, MAF = 0.09) is situated in the downstream region of the same gene. In a study by Lanubile et al. (2015), RNA sequencing was employed to investigate the molecular aspects of the interactions between a partially resistant genotype and FO36 (non-pathogenic) and FO40 (pathogenic) *F. oxysporum* isolates at 72 h post-inoculation. They observed that the expression of this gene increased to a certain extent compared to healthy controls during the same time period, with a fold change of approximately 3.4-fold for FO36 and 2.2-fold for FO40. Similarly, in this study, we found that the expression level of *Glyma.06G088400* in ZDD00774 (HR) also increased to a certain extent, reaching an approximate change of 9.7-fold at 72 h after treatment [10]. These findings suggest that *Glyma.06G088400* is a strong candidate gene that may play crucial roles in the response of soybean roots to *F. oxysporum* infection. However, further investigations and biological studies are needed to elucidate the specific mechanisms by which this candidate gene, with its LRR-RLK domain, participates in the plant defense response following *F. oxysporum* infection. Despite this need for further research, our results demonstrated the effectiveness of GWAS analysis in mapping soybean FORR resistance genes and provided the SNP markers that are tightly associated with FORR resistance. Moreover, these markers can be valuable tools for MAS.

Nearly all living organisms possess sensory proteins that enable them to detect environmental signals and transmit them within cells [38]. Among these proteins, LRR-RLKs play pivotal roles in plant development, growth, and stress responses [39–41]. Typically, the LRR-RLK gene family in plants consists of three functional domains: the N-terminal extracellular domain responsible for signal perception, the transmembrane domain that anchors the protein within the membrane, and the intracellular kinase domain involved in protein interactions and phosphorylation within downstream signal pathways [40, 42, 43]. Remarkably, Zhou et al. (2016) identified a total of 467 putative LRR-RLK genes, representing the largest LRR-RLK gene family discovered in plants at that time, with soybean harboring a substantial number of these genes [44]. Among these genes, 28 LRR-RLK genes are located on Chr 06, while *Glyma.06G088400* on Chr 06 is positioned distantly from neighboring LRR-RLK genes (*Glyma.06G086800*-126kb-*Glyma.06G088400*-190kb-*Glyma.06G090700*), constituting an independent region without tandem duplication clusters with other LRR-RLK genes [44]. This specific resistance gene presents an opportunity for plant breeders to more readily introgress and combine these genes in one breeding line. Consequently, it possesses considerable application value in soybean breeding programs aiming to enhance resistance against diseases and insects. Additionally, genetic diversity analyses conducted by Zhou et al. (2016) on 21 wild soybeans and 35 cultivated soybeans revealed intriguing findings regarding the *Glyma.06G088400* gene [45, 46]. They observed that the genetic diversity of *Glyma.06G088400* in cultivated soybean populations was estimated to be approximately 0.15 on average, significantly lower than that in wild soybean populations (approximately 0.49). Moreover, the average FST value (0.28) for *Glyma.06G088400* loci exceeded 0.15, indicating that, compared to their wild ancestors, cultivated soybeans experienced a decrease in genetic diversity and underwent selection pressures [44]. Given this disparity, it is anticipated that the FORR resistance identified in wild soybean populations may provide novel and valuable traits for swiftly reducing *F. oxysporum* damage and curbing the spread of the disease in soybean varieties. These significant findings provide valuable insights for breeding programs, directing future efforts towards enhancing *F. oxysporum* resistance in elite soybean cultivars by exploiting the genetic resources present in wild soybean germplasm. As the cost of acquiring high-density genome-wide marker sets decreases and GWAS statistical methodologies continue to advance, future challenges lie in developing precise, cost-effective, high-throughput, and accurate phenotyping methods. Overcoming these challenges could usher in a new era of crop disease management and plant breeding [47, 48].

## Conclusions 

In this study, GWAS was employed to identify SNP markers associated with FORR resistance, leading to the discovery of eight SNPs significantly linked to this trait. Notably, the expression of *Glyma.06G088400* showed differential patterns between highly susceptible and highly resistant accessions upon *F. oxysporum* infection, making it a potential candidate gene of interest. Further investigation of this gene, which potentially plays a crucial role in *F. oxysporum* resistance, is deemed necessary. The identification of these putative candidate genes and their corresponding significant SNPs holds great promise for unraveling the molecular mechanisms underlying FORR resistance and facilitating MAS in disease breeding programs aimed at developing soybean varieties with enhanced FORR resistance.

## Materials and methods

### Plant materials and *F. oxysporum* isolates

The 350 SGAs utilized in this experiment were sourced from the National Genebank of China (Beijing, China) and maintained by the Soybean Institute of Jilin Academy of Agricultural Sciences (Additional File 1: Table S1). These accessions consisted of 138 bred varieties (lines) and 212 landraces (Additional File 7: Table S6) mainly from Northeast China. The tests were conducted in the greenhouse facilities of the Jilin Academy of Agricultural Sciences (Gongzhuling, China) in September 2021. The *F. oxysporum* (FO_DH-A1-9) isolates used in this study was isolated and identified from the roots of diseased soybean plants in the Dunhua soybean field located in Jilin Province, China. The isolates were then stored on potato dextrose agar (PDA) medium.

### Inoculum preparation

*F. oxysporum* inocula were prepared by incubating them in PDA (26g Difco PDA/L) supplemented with antibiotics (0.10 g/L of ampicillin and 0.10 g/L of streptomycin sulfate) dishes at 25°C in the dark for one week. Sterile sorghum kernels (*Sorghum bicolor* (L.) Moench) were used, which were sterilized by soaking in distilled water overnight and autoclaved in quart mason jars (500 g seeds/jar) for two consecutive days, each time for 60 min. Each jar containing sterile sorghum kernels was then inoculated with 10 pieces of 7-day-old mycelial plugs from each individual *F. oxysporum* isolate. The jars were incubated at 25°C in the dark for two weeks to allow for the infection of the sorghum kernels. The jars were shaken for 3 to 5 min daily to ensure uniform fungal growth. For the nutrient substrate, a mixture of fine-grained soil and sand in a 1:1 volume ratio (V:V) was sterilized under high pressure at 121°C for 30 min, allowed to cool, and then mixed with the crushed and infected sorghum kernels. The ratio of infected sorghum kernels to sterilized sand-soil mixture was 1:50 (V:V), and thorough mixing was done until they were completely blended. Healthy soybean seeds were selected and treated with a 1% NaClO solution for 2 min, followed by treatment with 75% alcohol. The seeds were rinsed with sterile water for 2 to 3 times. These treated seeds were placed in plastic petri dishes containing moist sterile filter paper to ensure proper moisture and germination for one day. From each SGA, as well as the resistant and susceptible controls, four seeds were planted in seedling bowls (250 mL) filled with the inoculum mixed soil-sand mixture. After emergence, the seedlings were thinned to three plants, and each treatment was replicated six times. The experimental design employed was a randomized complete block design. The inoculated soybeans were maintained in a greenhouse with a photoperiod of 8 h of darkness and 16 h of light. The temperature in the greenhouse ranged from approximately 18 to 25°C, corresponding to night and day temperatures, respectively.

### Resistance evaluation in greenhouse

The disease reaction of soybean roots to *F. oxysporum* was evaluated four weeks after inoculation. The evaluation criteria for resistance in this study were modified from the method described by Chang et al. (2018) [7]. Each individual plant in the seedling bowls was assessed separately using a rating scale ranging from 0 to 4, where: 0 = no observable root symptoms, 1 = normal plant growth normally with a slightly brown taproot and well-developed fibril roots, 2 = significant growth impairment in the aboveground section, with predominantly brown taproot and noticeable brown spots on fibril roots, 3 = severe suppression of aboveground growth, completely brown taproots, and apparent brown spots on fibril roots, and 4 = plant death, with a dark brown, fractured taproots and reduced, brown fibril roots (Additional File 8: Figure S2). A DSI was calculated for each accession using the following formula: DSI = (Σ (rating of each plant)/4 × total number of plants rated) × 100. DSI data were obtained by investigating the disease grades of the 18 plants. The DSI values ranged from 0 to 100, representing the absence of disease symptoms to full coverage by the fungus (*F. oxysporum*). Based on the DSI, the FORR resistance of all SGAs was classified into the following categories: highly resistant (HR, 0 ≤ DSI < 25), resistant (*R*, 25 ≤ DSI < 40), moderately susceptible (MS, 40 ≤ DSI < 55), susceptible (S, 55 ≤ DSI < 70), or highly susceptible (HS, 70 ≤ DSI < 100).

### DNA extraction, genotyping, and quality control

Genomic DNA was extracted from fresh young leaves of soybean using the previously reported hexadecyl trimethyl ammonium bromide method [49]. Genotyping of all 350 SGAs was performed using the Illumina SoySNP50k iSelect BeadChip (Illumina, San Diego, Calif. USA), which includes 52,041 SNPs [50]. The International Union of Pure and Applied Chemistry (IUPAC) standard codes for nucleotides were used to represent the SNP data. Each SNP’s quality was individually examined, as previously described [51]. Missing SNP genotypes in the filtered dataset were imputed using beagle software [52]. SNPs lacking physical position information and displaying low quality (missing data < 20% and/or minor allele frequency (MAF) < 0.05) across all samples were excluded from the dataset. The remaining set of high-quality SNPs, totaling 30,602, was retained for further analysis.

### Population structure and linkage disequilibrium

Population stratification was assessed using PCA, neighbor-joining (NJ) trees, and population structure analysis. The PCA and evaluation of the kinship matrix were conducted using Tassel V5.2.60 software, based on 30,602 SNPs from the 350 SGAs. The NJ phylogenetic tree was constructed using the Maximum Composite likelihood model in MEGA-X, with 1000 replicates of Bootstrap values. Linkage SNP filtering was performed using PLINK V1.09, with a window size of 50 kb, SNP step size of 10, SNP correlation threshold of 0.2, and retention of unlinked SNPs. This resulted in a subset of 1643 SNPs for inferring population structure using STRUCTURE 2.3.4 [30]. The number of subgroups (K) was set from 1 to 10, with 5 replications. The burn-in period was set to 10,000, and the number of Monte Carlo Markov Chain replications was set to 100,000, with other options using the default values of the software. The most probable number of k was determined by analyzing the results with Structure Harvester [53], using the Delta K method described by Evanno et al. (2005) [54]. The estimation of pairwise LD was conducted on 30,602 SNPs using squared allele frequency correlations (*r*^2^) with PLINK1.09. Subsequently, mean LD decay plots were generated using an R script [55], plotting *r*^2^ values for SNPs with pairwise distances below 500 kb in either euchromatic or heterochromatic regions of each chromosome against physical distance. The chromosomal distance at which the mean *r*^2^ declined to half of its maximum value was used to calculate the LD decay rate relative to the population [56]. LD analysis and identification of haplotype blocks for significant SNPs were conducted using LDBlockShow Software [57].

### GWAS

A total of 30,602 SNPs from 350 SGAs were employed in the GWAS analysis to identify associations between SNPs and DSI. The GWAS analysis was conducted using GAPIT with GLM, MLM [58], CMLM [59], and SUPER [60]. In these analyses, the reduced Q and K matrices were included as covariates to account for population structure and familial relatedness, respectively. The observed *P*-values resulting from SNP-trait associations, along with the expected *P*-values assuming no associations between SNPs and traits, were used to generate Q-Q plots depicting the estimated log10(*P*) values. To mitigate the confounding effect of population structure, the model with observed *P*-values that were closest to the expected *P*-values was selected. The presence of significant association signals was determined using the Bonferroni threshold, with a threshold set at *P* ≤ 1/30,602, or -Log10(*P*) ≥ 4.49 [61].

### Candidate gene prediction

To identify the candidate genes underlying the association signals, we focused on significant SNPs linked to large-effect quantitative trait nucleotides (QTNs) and conducted a targeted search within their genomic regions. The candidate regions were defined based on either the mean LD decay distance or the LD block. For gene identification, we obtained functional annotations of gene models (Glyma.Wm82.a2.v1) or known genes located within the target genomic regions from the Soybase Database (http://www.soybase.org/). By leveraging the soybean genome annotations, we predicted potential candidate genes associated with the identified regions. Furthermore, the functional annotations of genes located in the target genomic regions were retrieved from Phytozome (http://www.phytozome.net).

### Production of the inoculum and inoculation procedure, and qRT-PCR assay

The inoculum for FO_DH-A1-9 isolates was cultivated on PDA for one week at 25°C in the dark. Conidia collection was performed through a meticulous process involving the thorough cleaning of the Petri dish with sterile water, followed by the scraping of the mycelium from the surface of the agar using an aseptic toothpick. Furthermore, the conidia suspension was filtered through sterile cloth and Miracloth to obtain the desired conidia. The concentration of FO_DH-A1-9 spore suspension was adjusted to 1 × 10^6^ cfu/ml based on microscopic counts using a Bürker chamber. Prior to inoculation, seeds of ZDD06827 (HS, DI = 100.0) and ZDD00774 (HR, DI = 0.0) were disinfected following previously described methods. Then, the disinfected seeds were uniformly distributed in a germination machine and stored in a growth chamber at 25°C, 80% relative humidity, and a dark environment for cultivation. Soybean seedlings ZDD06827 and ZDD00774 exhibiting good growth were selected and placed on germinating paper soaked in aseptic distilled water. They were subsequently inoculated with 200 μl of 1 × 10^6^ conidial suspension of FO_DH-A1-9 isolates using a pipette, while a control group received an inoculation of 200 ml of sterile water. The seedlings, along with the control, were placed vertically in a sterile 1000 ml beaker, and 300–500 ml of sterile water was added. The beaker was then kept in a growth chamber with conditions set at 25°C, 90% relative humidity, and a photoperiod of 16 h of light and 8 h of darkness.

Primary roots were sampled at 0, 6, 12, 24, 48, and 72 h after inoculation, and total RNA was extracted using the Easy Pure Plant RNA kit (QUANSHIJIN, China). Reverse transcription was performed using the PrimeScriptTM RT Reagent kit with gDNA Eraser (Takara, Japan) following a standardized protocol, with 1 μg of DNase-treated RNA. The qRT-PCR primers were designed using the Oligo7 software (Additional File 9: Table S7), and the housekeeping gene actin was selected as the control gene. The RT-PCR analyses were conducted to determine the expression levels of the candidate genes. RT-PCR amplifications were performed on the CFX48 ECO™ Real-Time PCR System (Illumina, USA) utilizing the RT-PCR kit according to the manufacturer’s instructions (Takara, Japan). The qRT-PCR reaction mixture was prepared by combining 0.2 µM primer premix, 2 µL of cDNA synthesis solution, 5 µL of TB Green Premix Ex Taq II (TaKaRa, Japan), and adjusting the final volume to 10 µL with ultra-pure water. The qRT-PCRs were performed as follows: 50°C for 2 min, 95°C for 3 min, followed by 40 cycles of 95°C for 10 s, 52 or 55°C (depending on the gene), and 72°C for 30 s. Three independent biological replicates were conducted to ensure accurate statistical analysis, and the relative expression levels of the candidate genes were evaluated using the comparative 2^−ΔΔCt^ method [62].

### Data analysis

Phenotypic data were evaluated and recorded using Microsoft Office Home and Student 2019 software. Differential saliency, correlation, and descriptiveness analyses were performed with SPSS software (version 22.0; IBM Corp, Armonk, NY, USA) [63]. Histograms, scatter plots, boxplots, and violin plots were generated using GraphPad Prism 9.2 software (GraphPad Company, San Diego, CA, USA). The normal distribution conformity of the phenotypic data was assessed through the Shapiro–Wilk test.

### Supplementary Information

**Additional file 1: Table S1.** Bioassay phenotypic results of resistance to FORR in 350 soybean germplasm accessions.

**Additional file 2: Figure S1. **Frequency distribution plot and Q-Q plot of DSI of 350 SGAs. (A) The DSI frequency distribution plot of 350 SGAs. (B) The Q-Q plot fitted by the correlation between the DSI of 350 SGAs and the normal distribution.

**Additional file 3: Table S2.** Summary of the polymorphic markers on the 20 chromosomes of *Glycine max*.

**Additional file 4:** **Table S3.** The Geographical distribution of 350 soybean germplasm accessions from different subgroups.

**Additional file 5: Table S4.** The Gene Ontology (GO) enrichment analysis of the candidate genes.

**Additional file 6: Table S5.** The eukaryotic orthologous groups (KOG) annotated analysis of the candidate genes.

**Additional file 7: Table S6.** Geographical sources of 350 soybean germplasm accessions.

**Additional file 8: Figure S2. ***F. oxysporum* root rot scoring scheme. Scoring scheme of soybean *F. oxysporum* root rot. Pictures (A–E) displayed phenotypes with varying disease severity ratings for the soybean root rot, including 0, 1, 2, 3, and 4.

**Additional file 9: Table S7.** Primer sequences used for expression profiling.

## Data Availability

The supporting data for this study are available in the CNGB Sequence Archive (CNSA) of the China National GeneBank DataBase (CNGBdb) under accession number CNP0005015 (https://db.cngb.org/).

## Abbreviations

FORR: *Fusarium* Oxysporum root rot
DSI: Disease severity index
SGAs: Soybean germplasm accessions
HS: Highly susceptible
S: Susceptible
MS: Moderately susceptible
R: Resistant
HR: Highly resistant
HLJ: Heilongjiang province
JL: Jilin province
LN: Liaoning province
IM: Inner Mongolia
BJ: Beijing
SX: Shanxi province
SNP: Single-nucleotide polymorphism
NJ: Neighbor-joining
GWAS: Genome-wide association study
QTNs: Quantitative trait nucleotides
LD: Linkage disequilibrium
PCA: Principal component analysis
Q-Q: Quantile–quantile
qRT-PCR: Real-time quantitative PCR
Chr: Chromosomes
GLM: General linear model
MLM: Mixed linear model
CMLM: Compressed mixed linear model
SUPER: Settlement of MLM under progressively exclusive relationship
LRR-RLK: Leucine-rich repeat receptor-like kinase
GO: Gene ontology
MAF: Minor allele frequency
MAS: Marker-assisted selection
