# Supplementary material for: Genome-wide association study on resistance of cultivated soybean to Fusarium oxysporum root rot in Northeast China
Source: BMC Plant Biol. 2023 Dec 7;23:625. doi: 10.1186/s12870-023-04646-5 (PMC10702129; doi:10.1186/s12870-023-04646-5)
Supplement: Supplementary file 2 — Additional file 2: Figure S1. Frequency distribution plot and Q-Q plot of DSI of 350 SGAs. (A) The DSI frequency distribution plot of 350 SGAs. (B) The Q-Q plot fitted by the correlation between the DSI of 350 SGAs and the normal distribution. [file 12870_2023_4646_MOESM2_ESM.docx]

**Additional file 2: Figure S1** Frequency distribution plot and Q-Q plot of DSI of 350 SGAs. (A) The DSI frequency distribution plot of 350 SGAs. (B) The Q-Q plot fitted by the correlation between the DSI of 350 SGAs and the normal distribution.
