# Supplementary material for: Genome-wide association study on resistance of cultivated soybean to Fusarium oxysporum root rot in Northeast China
Source: BMC Plant Biol. 2023 Dec 7;23:625. doi: 10.1186/s12870-023-04646-5 (PMC10702129; doi:10.1186/s12870-023-04646-5)
Supplement: Supplementary file 8 — Additional file 8: Figure S2. F. oxysporum root rot scoring scheme. Scoring scheme of soybean F. oxysporum root rot. Pictures (A–E) displayed phenotypes with varying disease severity ratings for the soybean root rot, including 0, 1, 2, 3, and 4. [file 12870_2023_4646_MOESM8_ESM.docx]

**Additional file 8: Figure S2** *F. oxysporum* root rot scoring scheme. Scoring scheme of soybean *F. oxysporum* root rot. Pictures (A–E) displayed phenotypes with varying disease severity ratings for the soybean root rot, including 0, 1, 2, 3, and 4.
